# Nutrition and Metabolism in the First 1000 Days of Life

**DOI:** 10.3390/nu15112554

**Published:** 2023-05-30

**Authors:** Yalin Zhou, Yajun Xu

**Affiliations:** 1 Department of Nutrition and Food Hygiene, School of Public Health, Peking University, No. 38 Xueyuan Road, Beijing 100083, China; zylyingyang@163.com; 2 Beijing Key Laboratory of Toxicological Research and Risk Assessment for Food Safety, Peking University, No. 38 Xueyuan Road, Beijing 100083, China; 3 PKUHSC-China Feihe Joint Research Institute of Nutrition and Healthy Lifespan Development, No. 38 Xueyuan Road, Beijing 100083, China

The first 1000 days of life are a critical window period for rapid growth and development during which individuals are more sensitive to the stimulation of environmental factors. Environmental stimuli at this stage not only affect health outcomes in early life but also have long-term effects on health trajectories later in life. Nutrition in early life, as an important and modifiable environmental factor, is closely related to fetal programming in utero, the growth and development of infants after birth and adult metabolic health [1]. This Special Issue, “Nutrition and Metabolism in the First 1000 Days of Life”, covers related studies on the effects of maternal and infant nutrition, infant feeding and supplementary food on pregnancy outcomes, as well as infant growth and development. The findings reported in this Special Issue provide a scientific basis and support the formulation of precise nutrition intervention strategies to improve maternal and infant health.

Maternal nutrition in early life has a direct and profound influence on fetuses and infants. During gestation, maternal nutrition affects fetal growth and development via the mother-to-fetus interface. Folic acid and choline, essential nutrients during pregnancy, are involved in the synthesis of nucleotides and phospholipids, as well as methylation [2]. 5,10-methylenetetrahydrofolate dehydrogenase (MTHFD1) is the key enzyme in folic acid metabolism and participates in choline metabolism. The most common MTHFD1 R653Q variant is the risk factor for neural tube defects (NTDs), intrauterine growth restriction and pregnancy loss [3]. Women carrying the MTHFD1 R653Q variant have an increased nutritional need for choline [2]. In this Special Issue, Christensen et al. [4] examined MTHFD1-synthetase-deficient mice fed choline-deficient diets to mimic the status of the MTHFD1 R653Q variant and mild choline deficiency to explore the effects of the interactions between them on pregnancy outcomes. They reported that the maternal MTHFD1 R653Q variant and mild choline deficiency altered maternal one-carbon metabolism and increased the risk of birth defects [4]. These findings highlight the role of gene–nutrition interactions in birth defects in humans and provide evidence for the development of precise individualized intervention strategies for nutrition during pregnancy. Hemoglobin (Hb) level is one of the assessment indicators of the nutritional status of pregnant women. Hb concentration during the trimesters has been reported to be associated with birth outcomes such as being small for gestational age (SGA); however, the conclusions are not consistent [5]. In the Special Issue, Xu et al. [6] revealed the association of maternal longitudinal hemoglobin with SGA during pregnancy and reported that women at low risk of iron deficiency, both higher Hb concentrations in late pregnancy and lower Hb reduction during pregnancy, were more susceptible to SGA. GDM is a prevalent metabolic disorder among pregnant women and greatly increases the risk of chronic non-communicable diseases of offspring. However, the potential mechanisms remain unclear. In this Special Issue, Zhou and colleagues [7] established the GDM animal model and adopted metabolomics to explore the altered metabolite profiles in the amniotic fluid and serum of GDM mothers and their correlation with maternal and fetal health outcomes, and finally identified the differentiate metabolites in the serum of pregnant women as biomarkers for predicting the intrauterine environment and fetal health status. The researchers also revealed the role of gut microbiota in the development of GDM and the relevant adverse pregnancy outcomes [8].

Lactation is the second critical stage in the first 1000 days of life. During this period, human milk is the main source of nutrition for infants and meets almost all the nutritional demands of infants within 6 months. Initiation time and duration of breastfeeding have profound beneficial impacts on maternal and infant health [9]. In this Special Issue, Shen et al. [10] reported that early initiation of breastfeeding (EIBF) contributed to a reduction in the risk of postpartum depression symptoms, extending breastfeeding duration and promoting exclusive breastfeeding. In terms of infants, EIBF is a protective factor preventing morbidity and mortality. The benefits of breastfeeding mainly depend on human milk composition. A great number of nutrients and bioactive substances in human milk are closely related to intestinal health, immunity and neurodevelopment of infants. Human milk is not only a liquid, but also a dynamic metabolic system containing a variety of small molecular metabolites [11]. Zhang and colleagues [12] detected these metabolites in human milk from the Chinese Human Milk Project (CHMP) cohort, and for the first time classified it into different metabotypes according to low-molecular metabolite profile in human milk, metabotype 1 and metabotype 2. They reported that metabotype 1 was associated with slow growth and development of infants, and metabotype 2 increased the risk of allergy in infants. Additionally, researchers also found that metabotypes were affected by maternal BMI. Therefore, it is of practical significance to predict the allergy risk of infants using different metabotypes of human milk. However, the metabotypes of human milk remain verified in a larger sample size of the population, and more studies are needed to clarify the potential mechanisms of the effects of human milk metabolites on infant health. With the deepening of research on the composition of breast milk, more and more active ingredients in breast milk have been mined, among which human milk oligosaccharides (HMOs) have been the cumulative focus. HMOs are the third solid component of human milk next to lactose and fat. As effective prebiotics, HMOs shape the structure and composition of infants’ gut microecology in early life. There are great individual differences in HMOs, and their contents and composition are affected by genetic background, geographical location, race and maternal health, as well as diet [13]. In this Special Issue, Zhou et al. [14] conducted a systematic review and meta-analysis, and reported for the first time the contents and dynamic changes in 14 typical HMOs in the milk of lactating mothers in China, and systematically compared the contents of representative HMOs in the milk of Chinese lactating mothers, such as 2′ FL, 3′ SL, etc., with those of lactating mothers in other countries. Studies on human milk sensory properties are increasingly gaining widespread attention. Human milk flavor plays a crucial role in successful breastfeeding. In this Special Issue, Yu et al. [15] reported, for the first time, the flavor characteristics and flavor substance composition of Chinese lactating mothers using the CHMP cohort, and constructed a three-tiered human milk flavor wheel based on sensory descriptors to achieve the qualitative and quantitative evaluation of human milk flavor. In this Special Issue, studies on small molecular metabolites, HMOs and flavor substances in human milk provide important scientific clues for deepening the understanding of human milk, promoting rational feeding and composing the infant formula to simulate human milk. 

Exclusive breastfeeding is recommended for infants for the first 6 months. However, infant formula becomes a viable alternative when breastfeeding cannot be performed due to a variety of reasons. To ensure the nutrition requirements of non-breastfed infants, researchers are committed to the optimization of infant formula composition based on human milk. Nevertheless, the marked differences between them mean that there is still a long way to go. For example, the protein content and composition of infant formula are significantly different from that in human milk, which is one of the main reasons for the higher growth velocity of formula-fed infants. There is no consensus on what proportion of protein is conducive to achieving normal growth and development in parallel with that of breast-fed infants. In this Special Issue, Ren and colleagues [16] conducted a systematic review and meta-analysis to comprehensively compare the relationship between different protein/energy ratios in infant formula and the growth of infants, and reported that the growth outcomes of infants fed with infant formula with a relatively low protein/energy ratios were comparable to those of breastfed infants. Complementary foods provide another source of nutrition for infants over 6 months [17]. Kittisakmontri et al. [18] found that protein content and sources of complementary foods affect physical growth, weight gain and growth-related hormone levels in infants through meta-analysis. In addition, Ye et al. [19] used metabonomics to figure out the correlation between protein metabolites and neonatal bronchopulmonary dysplasia. All of the above-mentioned findings suggest that the refinement of dietary protein pattern is beneficial to the healthy growth and development of infants and young children. However, the components in human milk, infant formula and complementary foods are complex and diverse, and the nutrients interact with each other [20]; thus, tremendous amounts of effort and numerous studies are still required to determine the most suitable dietary pattern for infant growth and development.

To summarize, during gestation, the maternal nutritional status reshapes the intrauterine environment and influences fetal programming. Deficiency or excess in essential nutrients such as folic acid and iron increases the risk of adverse pregnancy outcomes. The existence of SNPs related to nutrient metabolism emphasizes the vital role of nutrient-gene interaction in pregnancy outcomes. Within the first 2 years after birth, infant nutrition is provided by human milk, infant formula and complementary foods. The bioactive components in human milk represented by HMOs are strongly linked to infant growth. Research on functional components and sensory properties of human milk is fundamental to the optimization of infant formula composition. The omics technology facilitates the examination of functional components in human milk. Furthermore, rational infant complementary food patterns are conducive to the maintenance of normal infant growth. In conclusion, maternal and infant nutrition still requires extensive and sustained attention. In the future, more high-quality studies on nutrition and metabolism in the first 1,000 days of life should be carried out to promote maternal and infant healthcare, the progression of the disease intervention window period and the achievement of healthy development throughout the life cycle. 
